# Dengue and Scrub Typhus Co-infection at a Tertiary Care Centre in Nepal: A Case Series in Pediatric Patients

**DOI:** 10.31729/jnma.8893

**Published:** 2025-02-28

**Authors:** Sriram KC, Bulbul Pradhan, Inesh Khanal, Aakripa Rani Shrestha

**Affiliations:** 1 Department of Pediatrics, Patan Hospital, Patan Academy of Health Sciences, Lagankhel, Lalitpur, Nepal; 2 Patan Hospital, Patan Academy of Health Sciences, Lagankhel, Lalitpur, Nepal

**Keywords:** *co-infection*, *dengue*, *Nepal*, *pediatrics*, *scrub typhus*

## Abstract

This case series highlights four pediatric patients with coinfection of dengue fever and scrub typhus, presenting at Patan Hospital. Dengue fever is caused by flavivirus transmitted by Aedes mosquitoes, and scrub typhus is caused by Orientia tsutsugamushi transmitted by Leptotrombidium mites, which share overlapping symptoms such as fever, headache, and abdominal pain, complicating the diagnosis. The cases were from different geographical regions of Nepal with varied presentation: from febrile illness to severe manifestations like meningoencephalitis and raised intracranial pressure. Two patients presented with hepatosplenomegaly, one with severe rash, and another with pneumonia-like symptoms. Laboratory findings confirmed coinfection, and management included IV antibiotics, antipyretics, and supportive management. Complications such as thrombocytopenia, transaminitis, and allergic reactions required interventions. This case series highlights the importance of early suspicion and treatment of coinfection to reduce prolonged hospitalization and prevent end-organ dysfunction, and mortality, especially in endemic regions.

## INTRODUCTION

Dengue is a tropical disease caused by flavivirus transmitted by *Aedes aegypti* and *Aedes albopictus* mosquitoes. Scrub typhus is a rickettsial tropical disease caused by *Orientia tsutsugamushi* transmitted by larval trombiculid mites of *Leptotrombidium*.^1^ The clinical presentation of patients with dengue fever can vary from malaise, fatigability to shock and multi organ dysfunction syndrome as a part of severe illness.^2^ The presentation of patients with scrub typhus can be fever, headache, cough, abdominal pain, generalized lymphadenopathy and presence of a pathognomic eschar.^3^

Due to overlap between symptoms and endemicity of scrub typhus in "tsutsugamushi triangle" of South Asia, there is often a diagnostic and therapeutic dilemma while approaching pediatric patients with acute febrile illness.^1,3-5^ Co-infection can result in life threatening complications such as meningitis, pancreatitis and multiple organ dysfunction syndrome.^6,7^ Here, we showcase four instances of dengue and scrub typhus co-infection presenting at Patan Hospital with varied clinical features.

## CASE 1

A 10 year-young male child from Galchhi, Dhading, presented to the pediatric referral clinic (PRC) with a chief complaint of fever for 5 days. Fever was acute in onset, continuous type with maximum recorded temperature of 103°F. The fever did not resolve on taking paracetamol. Fever was associated with headache, retro-orbital pain and vomiting. There were multiple episodes of vomiting three days prior to presentation; vomitus was non-projectile, non-bilious and contained undigested food particles. There was no history of abnormal body movements, skin rashes, chills, rigor, nasal or gum bleeding, cough, fast breathing or chest pain. On examination, the child was afebrile with stable vital signs. Respiratory, cardiovascular and neurological examinations were grossly intact.

Complete blood count with hematocrit, differential leucocyte count and platelet count, renal function test, liver function test, blood culture, random blood glucose and C-reactive protein (CRP) were sent. CRP was raised with a value of 48.92 mg/L and there was leukocytosis with lymphocyte predominance (L52, N43, M2, E3, B0) whereas other tests were within normal limits. Microbiological testing revealed he was positive for Dengue Non-structural 1 (NS1) Antigen and Scrub Typhus. He was admitted to the children ward with a diagnosis of dengue fever without warning signs with scrub typhus. He was managed with IV fluids, Injection Paracetamol, Injection Ondansetron and Injection Doxycycline. After completing the seven days course of Doxycycline, the patient was discharged with stable vitals and significantly improved clinical status. Danger signs were explained to the parents.

## CASE 2

A 14-year young female child from Bakaiya, Makwanpur, presented to the emergency department (ED) with chief complaints of acute onset of fever for 1 week which was intermittent with a maximum temperature of 102°F. The fever didn't subside on taking paracetamol. It was associated with generalised abdominal pain for 1 week which was mild in severity. There was no history of cough, chest pain, abnormal body movements or loss of consciousness, skin rashes, burning micturition, joint pain or swelling. For these complaints she had taken Tablet Cefixime 200 mg twice daily for four days from a local pharmacy but her symptoms didn't resolve. She then presented to Hetauda Hospital and she was referred to Patan Hospital from there. Examination revealed an ill-looking child with a temperature of 102°F, with stable other vital signs. Abdominal examination revealed tenderness on the right hypochondriac region with hepatosplenomegaly. Liver span was 12 cm and the spleen was palpable 2 cm below the left costal margin. Respiratory, cardiovascular and neurological examinations were normal.

Complete blood count with hematocrit, differential leucocyte count and platelet count, renal function test, blood culture, random blood glucose were sent which were normal. Liver function Tests showed transaminitis with SGOT= 139 IU/L, SGPT= 77 IU/L, ALP= 304 IU/L and CRP was 28.66 mg/L. Microbiological testing revealed Dengue NS1 Antigen and Scrub Typhus positive, one week after the onset of initial symptoms.

The patient was admitted to the Pediatric High Dependency Unit (PHDU) with a diagnosis of Scrub typhus with Dengue fever without warning signs and was treated with IV fluids, Injection Paracetamol and Injection Doxycycline. On the second day of admission, the patient developed features of raised Intracranial Pressure (ICP): right lateral rectus palsy, bradycardia and hypertension. Ophthalmological consultation revealed papilledema. The patient was transferred to the Pediatric Intensive Care Unit (PICU) & was managed with 3% Normal Saline (NS). During PICU stay, the patient developed thrombocytopenia (Platelet Count: 45,000/pl), so 3% NS was stopped and Inj. Mannitol was given as a stat dose. Prior to the second dose of Inj. Mannitol, she developed hypotension, so Mannitol was stopped and 3% NS was restarted as her platelet counts had normalised to 154,000/pl. Ophthalmological review revealed no sign of papilledema on fifth day of admission. Her cerebrospinal fluid was then sent for analysis. It revealed RBC count: 14 per mm^3^, WBC count: 2 per cumm, DLC: L2 and PMN:00, Protein 152.5 mg% and Sugar 52 mg%. The final diagnosis was Scrub typhus with scrub meningoencephalitis with features of raised ICP with Dengue fever. She was transferred to the children's ward and treated with IV antibiotics & was discharged on the ninth day of admission after she became clinically and hemodynamically stable. Danger signs were explained to the parents and kept on follow-up.

## CASE 3

A 4 years-young male from Melung, Dolakha presented to the ED of Patan Hospital with a high grade fever for 4 days with maximum recorded temperature of 104°F which was acute in onset, continuous in nature and not relieved by the use of paracetamol. It was associated with productive cough for the same duration and fast breathing for the past two days. There was no history of chest retraction, chest pain, abnormal body movement or loss of consciousness, skin rashes, burning micturition, joint pain or swelling. The child had received a single dose of syrup amoxicillin from a local pharmacy. Examination revealed a febrile child with a temperature of 102°F, with stable other vital signs. Respiratory examination revealed coarse crepitations in the right inframammary region. Cardiovascular, abdominal and neurological examinations were normal.

Complete blood count with hematocrit, differential leucocyte count and platelet count, renal function test, liver function test, blood culture, random blood glucose were sent which was normal. Chest X-ray revealed infiltration over the right lower zone & there was an elevated CRP level of 52.08 mg/L.

The child was admitted to the children ward with a diagnosis of Community Acquired Pneumonia (CAP) and was started on Inj ampicillin. The fever was persistently high till day three of admission, further workup was done and the antibiotic was changed to Inj Ceftriaxone. The fever remained consistently high till the sixth day of admission. So additional workup was done, which revealed Scrub Typhus and Dengue NS1 Ag positive. The diagnosis of Scrub Typhus with scrub pneumonitis with Dengue fever without warning signs was made & Inj Doxycycline was added but the patient developed an allergic reaction to doxycycline so it was replaced with syrup Azithromycin at 12.5 mg/kg/day. The fever still persisted after two days of azithromycin use so, blood culture was send and syrup chloramphenicol was added on the eighth day of admission after which the child became afebrile and clinically better. The culture report showed no growth and even tually, the child was discharged on the twelfth day of hospital stay, and advised to continue the syrup chloramphenicol for three more days and kept on regular follow-up.

## CASE 4

A 4 years-young male from Chichila, Sankhuwasabha presented to the PRC with fever for 2 weeks which was acute in onset, continuous in nature with maximum recorded temperature of 102°F. Fever was associated with skin rashes which was insidious on onset beginning from the left axillary region which later became generalised. Initially the rashes were macular, reddish, painful and non-pruritic; later the rash became progressively black in colour. There was no history of gum bleeding, passage of black tarry stool, loss of consciousness, abnormal body movement, cough, shortness of breath or burning micturition. The patient had initially presented to a health centre at Biratnagar from where he was referred to Patan Hospital. On examination, the child was ill-looking and lethargic, with a temperature of 100°F with stable other vital signs. Abdominal examination revealed non-tender hepatosplenomegaly with liver span of 9 cm and the spleen was palpable 2 cm below the left costal margin. Respiratory, cardiovascular and neurological systems were grossly intact.

He was treated with antipyretics and antibiotics in Biratnagar for two days but his symptoms didn't subside, so he was referred to Patan Hospital. Complete blood count with hematocrit, differential leucocyte count and platelet count, renal function test, liver function test, blood culture, random blood glucose and CRP were sent. He had a hemoglobin of 8.2 g/dl for which iron profile and peripheral blood smear (PBS) was done. Microbiological testing revealed Dengue NS1 Antigen and Scrub Typhus positive. Iron Profile and PBS showed iron deficiency anemia (IDA).

So, the child was admitted to the PHDU with a diagnosis of Scrub Typhus with dengue fever without warning signs with IDA. The patient received Inj. Doxycycline along with supportive management for seven days and was discharged after the child improved clinically.

## DISCUSSION

This case series highlights the clinical complexity and co-infection of Dengue fever and Scrub Typhus, both vector-borne illnesses, presenting in pediatric patients with overlapping symptoms and varying degrees of severity. All cases had high-grade fever that was unresponsive to antipyretics and antibiotics except for doxycycline, azithromycin, and chloramphenicol. Systemic involvement was present which manifested as meningoencephalitis with signs of raised intracranial pressure in the 2nd case, pneumonia with persistent fever in the 3rd case and hepatosplenomegaly in the 2nd and 4th case. There were variable laboratory findings like transaminitis, thrombocytopenia, and anemia in different cases.

In a retrospective analysis of dengue and scrub typhus co-infection diagnosed between January 2010 and July 2014 at a tertiary care center in South India, haemoglobin was normal with significant thrombocytopenia, transaminitis.^8^ In Cases 1, 2 and 3, the patients had normal hemoglobin whereas in Case 4, there was IDA. Dengue and scrub typhus infections typically do not cause significant changes in hemoglobin levels unless complicated by bleeding or hemolysis.^7^

In Case 2 the patient had thrombocytopenia and transaminitis. Thrombocytopenia is a hallmark feature of dengue, caused by direct viral suppression of megakaryocytes and increased peripheral destruction of platelets. It may also occur in scrub typhus due to endothelial injury and disseminated intravascular coagulation (DIC).^3,9^ Thrombocytopenia in both infections is immune-mediated so coinfected patients are likely to experience synergistic immune suppression of platelets, resulting in lower platelet counts.^10^ This was a contrasting finding as compared to our cases 1, 3 and 4, where the co-infected patients had normal platelet levels. Thrombocytopenia was only observed in Case 2.

As observed in Case 2, transaminitis is a common manifestation in both dengue and scrub typhus. In dengue, liver damage is caused by viral replication within hepatocytes and an immune-mediated inflammatory response.^9^ In scrub typhus, *Orientia tsutsugamushi* directly infects endothelial cells, causing vasculitis and hepatic inflammation.^9^ The co-existence of these conditions likely exacerbates hepatic dysfunction, emphasizing the importance of liver function monitoring.

In Case 3, there was a delayed diagnosis of Scrub Typhus and Dengue fever. As the symptoms were fever and cough, a provisional diagnosis of CAP was made and treatment was based on the provisional diagnosis. No improvement in the clinical findings despite the use of intravenous antibiotics is what eventually led to further workup which aided in the diagnosis of Scrub Typhus and Dengue co-infection. There can be wide range of clinical features of both scrub typhus and dengue fever.^11^ So, it is important to remain vigilant for such signs and symptoms, while managing a case of acute febrile illness especially in our part of the world.^2,3^

All the cases that were present were dengue NS1 positive. Regardless of the time duration, none of the cases were IgM positive. Cross-reactivity of NS1 antigen have been reported for Japanese encephalitis virus (JEV) and zika virus ZIKV. ^11^ Dengue virus, ZIKV and JEV are from the same genus flavivirus. Besides clinical features, the three viruses also share similarities of the NS1 antigen.^11^ Furthermore, ZIKV shares the same vectors as the dengue virus. Dengue fever and Scrub typhus differ in this regard. The do not share a common vector and their causative organisms are also different. So, positivity of nonstructural protein in absence of dengue infection and only in scrub typhus is unlikely unless there is another co-existing infection by a flavivirus.^12^

Lastly, clinicians should be watchful for the varying clinical presentations of both scrub typhus and dengue fever. Dengue fever can lead to plasma leakage, hemorrhagic manifestations, and organ failure whereas scrub typhus can lead to meningoencephalitis, pneumonitis, and hepatosplenomegaly.^9^ There have been case studies of dengue and scrub typhus co-infection that led to severe life-threatening complications like aseptic meningitis, meningitis, meningoencephalitis, septic shock, pancreatitis and MODS.^6,7^ Larger studies exploring the pathophysiology of co-infection and the impact on clinical outcomes are needed to guide evidence-based practices.

## CONCLUSIONS

The overlapping clinical and laboratory features of dengue and scrub typhus make early diagnosis and treatment of co-infections a significant challenge for physicians. Prompt suspicion of co-infection is crucial, as delays in diagnosis can result in prolonged hospital stays, an increased risk of complications and mortality.
